# 2C-ChIP: measuring chromatin immunoprecipitation signal from defined genomic regions with deep sequencing

**DOI:** 10.1186/s12864-019-5532-5

**Published:** 2019-02-28

**Authors:** Xue Qing David Wang, Christopher J. F. Cameron, Denis Paquette, Dana Segal, Reid Warsaba, Mathieu Blanchette, Josée Dostie

**Affiliations:** 1 Department of Biochemistry and Rosalind & Morris Goodman Cancer Research Center, McGill University, Montréal, Québec, H3G 1Y6 Canada; 2 School of Computer Science and McGill Center for Bioinformatics, McGill University, Montréal, Québec, H3A 0E9 Canada

**Keywords:** Chromatin immunoprecipitation, Next-generation sequencing, Ligation-mediated amplification, Epigenetics, *HOX*, Differentiation, Long non-coding RNA, Transcription, 5C, subTAD

## Abstract

**Background:**

Understanding how transcription occurs requires the integration of genome-wide and locus-specific information gleaned from robust technologies. Chromatin immunoprecipitation (ChIP) is a staple in gene expression studies, and while genome-wide methods are available, high-throughput approaches to analyze defined regions are lacking.

**Results:**

Here, we present carbon copy-ChIP (2C-ChIP), a versatile, inexpensive, and high-throughput technique to quantitatively measure the abundance of DNA sequences in ChIP samples. This method combines ChIP with ligation-mediated amplification (LMA) and deep sequencing to probe large genomic regions of interest. 2C-ChIP recapitulates results from benchmark ChIP approaches. We applied 2C-ChIP to the *HOXA* cluster to find that a region where H3K27me3 and SUZ12 linger encodes *HOXA-AS2*, a long non-coding RNA that enhances gene expression during cellular differentiation.

**Conclusions:**

2C-ChIP fills the need for a robust molecular biology tool designed to probe dedicated genomic regions in a high-throughput setting. The flexible nature of the 2C-ChIP approach allows rapid changes in experimental design at relatively low cost, making it a highly efficient method for chromatin analysis.

**Electronic supplementary material:**

The online version of this article (10.1186/s12864-019-5532-5) contains supplementary material, which is available to authorized users.

## Background

**Fig. 1:**
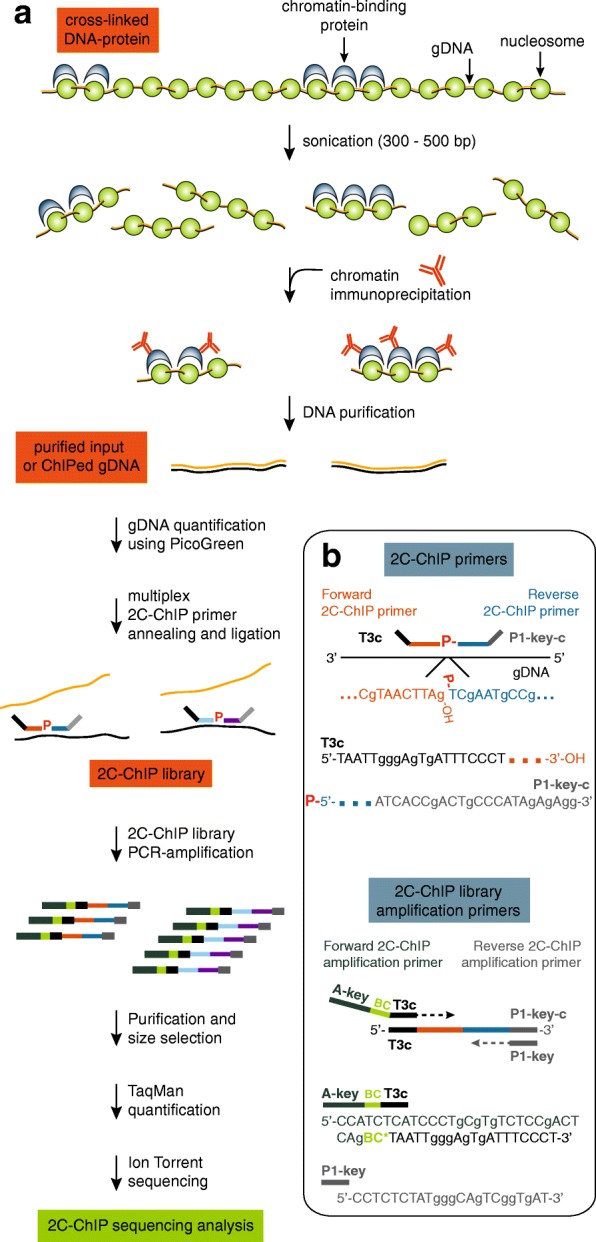
Carbon copy-chromatin immunoprecipitation (2C-ChIP) combines ligation-mediated amplification and next-generation sequencing to measure co-immunoprecipitated DNA from given genomic regions. **a** Overview of the 2C-ChIP method. Chromatin is first immunoprecipitated with desired antibodies, and the resulting purified genomic DNA or corresponding input is used to produce a 2C-ChIP library by annealing and ligating 2C-ChIP primers in a multiplex setting. Resulting libraries are then PCR-amplified with primers featuring barcodes and processed for sequencing. **b** Bottom right insert highlights primer design in 2C-ChIP analysis. Genomic regions are detected by ligating Forward and Reverse 2C-ChIP primers annealed to the same DNA strand. Note that Reverse primers must be 5’end-phosphorylated for ligation to occur. Primer design shown here is for sequencing on Ion Personal Genome Machine™ (PGM™) system (Thermo Fisher Scientific) but can be adapted for sequencing on any platform. Forward 2C-ChIP primers include a universal sequence of choice (here a T3 complementary sequence; T3c) at their 5′ ends. Reverse 2C-ChIP primers are 5′-phosphorylated and feature the complementary PGM™ P1-key sequence (P1-key-c) destined to bind the Ion Sphere Particles (ISPs) used in sequencing. The libraries are then PCR-amplified with “2C-ChIP library amplification primers” to incorporate barcodes. The Forward amplification primer includes – 5′ to 3′ – an A-key sequence used in the PGM™ system, the barcode (BC), and a T3 sequence. The Reverse amplification primer is the P1-key sequence. Sequencing will occur from the A-key sequence such that barcodes will be read first

Understanding why genes are expressed at a given time will require full knowledge of how transcription occurs. Acquiring this knowledge, in turn, will demand the integration of many different types of information, including genomic sequence, epigenomic traits, and three-dimensional chromatin organization. Epigenomic information can be gained from robust complementary methodologies, one of which is chromatin immunoprecipitation (ChIP), which is now considered a staple in gene expression studies. ChIP is a powerful technique used to map the association of proteins and their post-translational modifications on genomic DNA. This method typically uses formaldehyde cross-linking to reversibly secure proteins onto DNA (Fig. 1a, *top*). DNA-protein complexes are next sheared via sonication, and proteins of interest are selectively immunoprecipitated with specific antibodies. The resulting associated DNA fragments are ultimately purified before sequence and abundance are determined.

Since it was pioneered, the ChIP protocol [1–3] has been adapted to improve speed, work on a smaller cell number, and/or increase its specificity [4]. Detection of ChIP signal has similarly been subject to many changes from its original DNA hybridization readout. Whereas quantitative real-time polymerase chain reaction (ChIP-qPCR) with specific primers remains the method of choice when analyzing few pre-determined candidate target sequences, ChIP followed by deep sequencing with next-generation technologies (ChIP-seq) is most commonly used for genome-wide profiling [5].

ChIP-seq is a powerful approach that identifies binding sites across an entire genome to provide complete views of protein and/or modification landscapes. However, understanding transcription will not only require genome-wide information but also its integration with data collected from the comprehensive analysis of defined genomic domains. In fact, ChIP-seq is not always the appropriate method of analysis, particularly when studying defined genomic regions. Indeed, interest is often directed towards given genomic region(s), and as the scope of analysis is increasingly broadening to include either numerous time points or different forms of perturbation (e.g., RNA interference knockdown, CRISPR-Cas9 gene editing, drug treatment), the high cost of ChIP-seq can limit the number of cell samples to be profiled, how many proteins or modifications can be analyzed, and/or the depth at which profiles are generated in multiplexed samples. In such comprehensive studies, measuring individual DNA regions by ChIP-qPCR is also inappropriate even for relatively short regions as it would be far too labor-intensive. ChIP-qPCR also uses a considerable amount of ChIP material to measure individual DNA sequences, which severely limits what can be quantified in an experiment. Thus, there is a need for a new form of high-throughput ChIP analysis method.

Here we present a ChIP-based methodology for profiling large pre-defined genomic regions. We refer to this method as carbon copy-ChIP or ‘2C-ChIP’. This approach, outlined in Fig. 1, is inspired by the chromosome conformation capture carbon copy technique (5C; [6]). 2C-ChIP combines conventional ChIP with highly multiplexed ligation-mediated amplification (LMA) to selectively copy and amplify ChIP signal from desired genomic regions (Fig. 1b). The resulting 2C-ChIP libraries are then analyzed by quantitative, high-throughput DNA sequencing. Given the rather low complexity of 2C-ChIP libraries, products can be barcoded and further multiplexed before sequencing on next-generation instruments to provide very high depth and coverage of many different ChIP profiles in single 2C-ChIP samples.

We developed and validated the 2C-ChIP protocol by analyzing the *HOXA* gene cluster during retinoic acid (RA)-induced differentiation of human pluripotent NT2-D1 cells into neuroectodermal lineages as we have done previously [7]. Through this analysis, we recapitulated our previous data and identified a domain that is slow to lose its H3K27me3 signal, forms a subTAD when viewed by 5C-seq [8], and contains the *HOXA-AS2* long non-coding RNA (lncRNA) gene. *HOXA-AS2* is the second lncRNA gene activated during RA-induced differentiation. Interestingly, within this domain is a small region overlapping the *HOXA-AS2* gene that was previously shown to repress transcription ectopically and act as a polycomb repressive element (PRE) in drosophila [9]. We found that preventing the accumulation of *HOXA-AS2* during RA induction drastically reduces the expression of all the proximal *HOXA* genes. Lower H3K4me3 levels at all promoters accompanied these changes without affecting H3K27me3 level changes in the gene bodies. Importantly, 5C-seq revealed that curbing HOXA-AS2 accumulation via RNA interference led to greater contact frequencies within its own subTAD, and to lesser long-range interactions in the proximal region. Thus, these structural changes might contribute to lower *HOXA* expression levels by distancing enhancers and genes. Based on this 2C-ChIP analysis and its integration with chromatin conformation data, we suggest that *HOXA-AS2* contributes to the collinear *HOXA* activation by maintaining gene expression during the later stages of induction in part by controlling the cluster’s spatial organization.

## Results

The ChIP protocol, which is previously described in ([10]; http://younglab.wi.mit.edu/hESRegulation/Young_Protocol.doc), is outlined in Fig. 1a (*top*). A ChIP experiment typically yields a complex array of DNA sequences that are selectively immunoprecipitated from entire genomes in a population of cells (‘*purified ChIPed gDNA*’). The abundance of each sequence reflects the frequency at which the protein or modification is found at that position in the cell population. During analysis, sequence abundance is most commonly measured using a candidate approach with specific primers, or genome-wide by quantitative sequencing. In either case, a sample of the chromatin used for ChIP is included as control (‘*purified input gDNA*’).

### Overview of the 2C-ChIP technology

The 2C-ChIP method fills the need for a versatile, low-cost, and high-throughput approach to quantify ChIP signals at defined genomic regions. 2C-ChIP detects immunoprecipitated DNA sequences by multiplex ligation-mediated amplification (LMA; Fig. 1b). During LMA, primer pairs are annealed next to each other on the same DNA strand, and only contiguously annealed primers can be ligated to quantitatively detect target sequences. The 2C-ChIP primers feature universal tails such that products from individual libraries can be PCR-amplified in a single step. The amplification primers also contain barcode sequences, which allows multiplexing of the 2C-ChIP libraries themselves prior to sequencing. As with any other LMA-based approach ([6] and references therein), 2C-ChIP can be conducted with thousands of primers in single reactions to detect target sequences at genome-scale without compromising linearity.

To analyze target regions by 2C-ChIP, a mixture of adjacent forward and reverse primer pairs is first annealed to ChIPed material. As usual in LMA, only reverse primers are 5′ end-phosphorylated to enable ligation. The primer pairs are designed to tile the region(s) of interest at a density selected to achieve the coverage and resolution desired. The annealed primer pairs are next ligated with Taq DNA ligase, which selectively ligates hybridized oligos only when positioned next to each other without overlap or gap between them. This step effectively produces a quantitative imprint or “carbon copy” of the probed sequences in immunoprecipitated samples. The resulting 2C-ChIP libraries are then PCR-amplified with sequencing primers of the platform of choice, featuring barcodes that are designed against the universal tails of the 2C-ChIP primers (Fig. 1b).

### The *HOXA* gene cluster as a model system

**Fig. 2:**
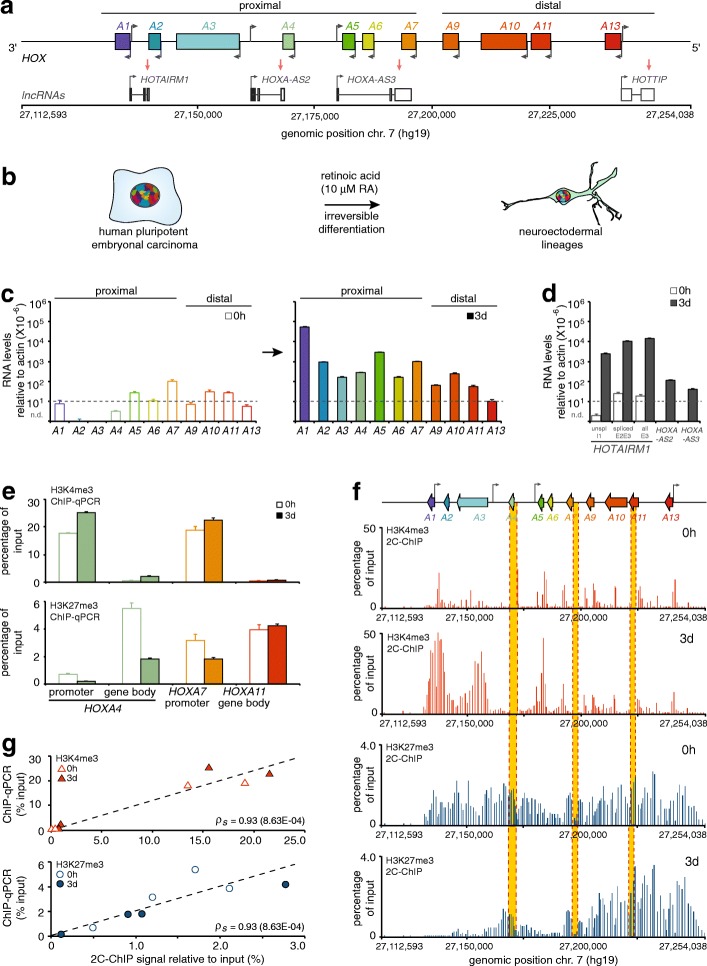
Results of 2C-ChIP analysis at the *HOXA* gene cluster correlate well with corresponding ChIP-qPCR data. **a** Schematic representation of the *HOXA* gene cluster included in the genomic region characterized in this study. The protein-coding *HOXA* genes are represented by rectangles with left-facing arrows to indicate gene orientation. Grey arrows pointing to the right show the transcriptional start site positions of long non-coding RNAs (lncRNAs) transcribed from the opposite strand. **b** Cell differentiation system used to develop and optimize the 2C-ChIP technique. Proximal *HOXA* (**c**) and *lncRNA* (**d**) genes are induced with RA. Steady state transcript levels measured by RT-qPCR are shown in (**c**) before (0 h; *left*), and after (3 days; *right*) cell treatment with 10 μM RA. LncRNA levels (**d**) are presented on the same graph. The *y-axis* shows RNA levels relative to actin where dashed lines indicate values below which measurements are unreliable. All RNA measurements are from at least three PCRs in each of two biological replicates (Additional file 2: Table S2). Error bars represent standard deviations. **e** ChIP-qPCR analysis of H3K4me3 and H3K27me3 changes occurring at select *HOXA* genes upon a 3-day RA treatment. Primer sequences are shown in Additional file 3: Table S3, and regions probed are highlighted in yellow in panel **f**. Error bars are standard deviations from at least 3 PCRs. **f** 2C-ChIP analysis of H3K4me3 and H3K27me3 at the *HOXA* gene cluster before and upon RA treatment for 3 days. Data shown here is limited to the region encoding genes and excludes most of the surrounding negative controls. Complete BED files are in Additional file 6: BED file 1–4. **g** Correlation between ChIP-qPCR results and corresponding 2C-ChIP signals for H3K4me3 and H3K27me3 ChIPs in uninduced and 3-day RA-induced NT2-D1 cells (Spearman’s rho = 0.93 for both assays)

We developed and validated the 2C-ChIP approach by characterizing the *HOXA* gene cluster region in NT2-D1 cells (Fig. 2a, b). The *HOX* genes are homeobox family members that encode helix-turn-helix transcription factors [11, 12]. In vertebrates, there are 39 *HOX* genes organized into 4 genomic clusters (*A*, *B*, *C*, and *D*) located on different chromosomes, with the human *HOXA* cluster residing on chromosome 7. *HOX* genes are highly conserved throughout evolution and play important roles both during development and adulthood.

During development, *HOX* genes are master regulators of anterior-posterior (A-P) body patterning, and are involved in organogenesis and the formation of limbs [13–16]. During body plan formation, *HOX* spatial and temporal expression is observed to follow the order of their positions along their respective chromosomes, with those at the cluster 3′ end expressed more anteriorly and earlier than those at the 5′ end. Similarly, during limb formation, 3′ end genes are expressed earlier and in the proximal limb region compared to those within the cluster’s 5′ end, which are turned on later and more distally in the limb. This spatio-temporal collinearity appears to emerge from many different types of control mechanisms, including the modulations of chromatin landscape and of three-dimensional chromatin organization. These underlying mechanisms have been scrutinized for over three decades and still remain under intense investigation.

An interesting feature of the human *HOXA* gene cluster is that it resides in a bivalent domain when transcriptionally silent [17]. The *HOXA* cluster contains 9 developmentally regulated protein-coding genes encoded on the same DNA strand and 4 intergenic lncRNAs transcribed from the opposite strand (Fig. 2a). Bivalent chromatin regions are DNA segments featuring both activating (H3K4me3) and repressing (H3K27me3) epigenetic marks. *HOX*, like many other genes that regulate cellular differentiation, are thought to exist in this state to keep them inactive but poised for rapid activation [18]. Whether transcription of a gene will be turned on appears to depend on the removal of polycomb group proteins (PcG) and their modifications [19]. Bivalent domains are also often regulated by lncRNAs that recruit enzyme complexes responsible for activating or repressing histone marks [7, 20, 21].

Interestingly, at least one of these lncRNAs can regulate three-dimensional chromatin organization as a mechanism to control the expression of genes. We recently demonstrated that HOTAIRM1 is required for proper collinear activation of the proximal *HOXA* genes by RA [7]. HOTAIRM1 is a lncRNA encoded between *HOXA1* and *2* that is rapidly induced upon cell differentiation by RA in NT2-D1 cells. It binds chromatin-modifying complexes, and prevents premature expression of *HOXA* genes during the early stages of induction. Indeed, preventing HOTAIRM1 accumulation by RNAi results in failure to physically uncouple the *HOXA1/2* and *HOXA5/6/7* subTADs, leading to the premature expression of the latter genes. Thus, analyzing *HOXA* induction by RA in NT2-D1 cells, which recapitulate the cluster’s temporal collinearity observed in development [22, 23], allows for the integration of 2C-ChIP results with that of three-dimensional chromatin organization, lncRNA activity, and gene expression.

### Validating 2C-ChIP against ChIP-qPCR and ChIP-seq data

The NT2-D1 cell model (Fig. 2b) is used extensively to study *HOX* gene regulation as it recapitulates their induction pattern in developing axial systems upon RA treatment [24–27]. Others and we have previously characterized the various changes in chromatin landscape accompanying this process [7, 26, 28, 29]. To develop 2C-ChIP with this differentiation system, we first measured gene expression in sample sets using quantitative real-time RT-PCR (RT-qPCR) (Fig. 2c, d; Additional file 1: Table S1) with previously validated primers ([7, 27, 30]; Additional file 2: Table S2). As expected [7], *HOXA* gene levels are nominal prior to induction and the expression of proximal genes is greatly increased upon RA treatment (Fig. 2c). LncRNA levels behave similarly, with HOTAIRM1 as the most induced transcript after the 3-day treatment (Fig. 2d). Using fixed chromatin, we then measured the levels of H3K4me3 and H3K27me3 by ChIP-qPCR at the promoter and within the body of strategically selected genes to assess the extent of epigenetic changes in matched sample sets (Additional file 3: Table S3). As expected [7], we find that H3K4me3 is present preferentially at the promoter of proximal genes before and after induction, and that it increases slightly following RA treatment (Fig. 2e, *top*). In contrast, H3K27me3 is found both at promoters and within gene bodies, and sharply decreases specifically at the proximal genes after RA induction (Fig. 2e, *bottom*).

Having verified that sample sets display the expected epigenetic changes upon treatment with the morphogen, we then used these fixed chromatin preparations to measure changes in H3K4me3 and H3K27me3 levels by 2C-ChIP (Fig. 2f). We designed 160 primer pairs covering the *HOXA* cluster at high density, as well as surrounding upstream and downstream regions at lower density to serve as negative controls (Additional file 4: Table S4; Additional file 5: Table S5). As would be expected from the ChIP-qPCR results, we observed higher levels of activating H3K4me3 at the promoter of proximal genes, accompanied with lower levels of H3K27me3 after 3 days of RA induction (Fig. 2f; Additional file 6: BED file 1–4). To compare the ChIP-qPCR and 2C-ChIP data, we calculated the average score of all 2C-ChIP probes located within a 1-kb window centered at the corresponding ChIP-qPCR amplicon, and calculated a Spearman-ranked correlation between the two data types. We find that the changes measured by 2C-ChIP correlate well with those evaluated by ChIP-qPCR (Fig. 2g). We also obtained similar ChIP-qPCR and 2C-ChIP results for SUZ12, a component of the polycomb repressive complex 2 (PRC2) responsible for the silencing H3K27me3 mark (Additional file 7: Figure S1; Additional file 6: BED file 5, 6).

**Fig. 3:**
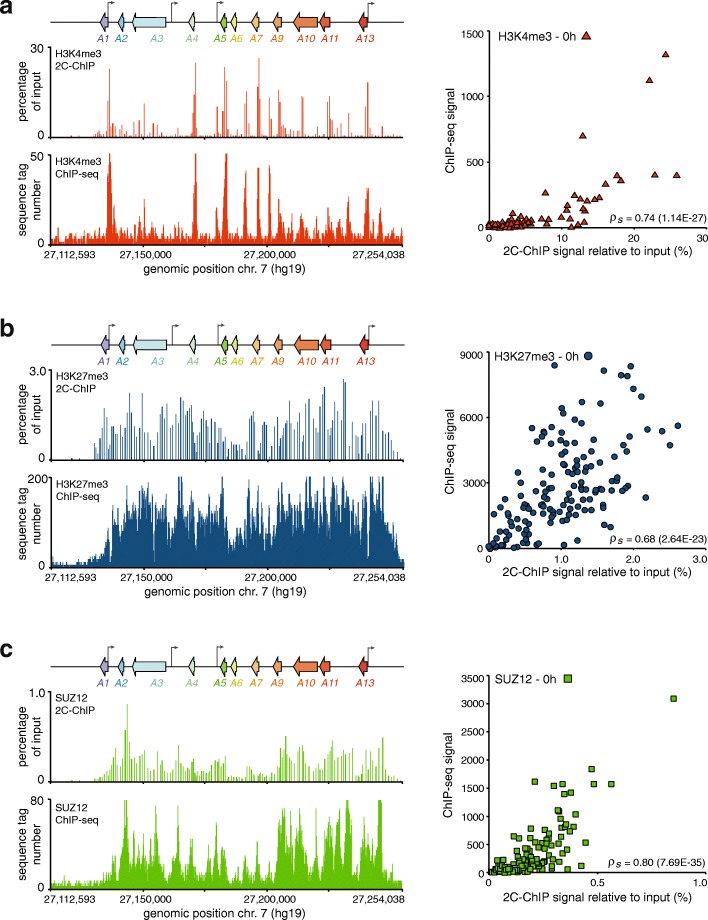
2C-ChIP and ChIP-seq results correlate well at the *HOXA* gene cluster. 2C-ChIP and ChIP-seq analysis of H3K4me3 (**a**), H3K27me3 (**b**), and SUZ12 (**c**) over the cluster region. Tracks seen on the left are limited to the gene-encoding region to highlight comparable detection of peaks and valleys by each assay. Complete 2C-ChIP BED files that include surrounding negative controls are in Additional file 6: BED file 1, 3, and 5. Correlation between 2C-ChIP and ChIP-seq results is found on the right of each panel and is between regions featured in both assays, excluding measurements equal to zero. Spearman’s rho is indicated on the bottom right of each graph

We next compared our 2C-ChIP profiles with available ChIP-seq datasets from untreated NT2-D1 cells where the *HOXA* cluster is transcriptionally silent, covered with H3K27me3, and gene promoters are marked with H3K4me3 (Fig. 3a, b, *left*). ChIP-seq data for SUZ12, which like the modification it catalyzes on histones covered the entire cluster region, was also available (Fig. 3c, *left*). With our primer design (Additional file 8: Figure S2a), 2C-ChIP recapitulated the distribution of histone marks and SUZ12 throughout the region. Similar patterns of peaks and valleys were observed with both approaches. In this case, to directly compare the two data types, we added all the ChIP-seq reads in the raw datasets spanning the corresponding probed 2C-ChIP sequences, and calculated the Spearman-ranked correlation. When quantified in this fashion, 2C-ChIP and ChIP-seq displayed strong positive correlations (H3K4me3: *ρ*_*s*_ = 0.74; H3K27me3: *ρ*_*s*_ = 0.68; SUZ12: *ρ*_*s*_ = 0.80), despite the fact that different antibodies were used and ChIP samples were prepared by different laboratories (Fig. 3a, b, c, *right panels*). Together, these results show that 2C-ChIP quantitatively recapitulates data obtained either by ChIP-qPCR or ChIP-seq, two current benchmark ChIP signal analysis approaches.

### Exploring the linear detection range of 2C-ChIP

The 2C-ChIP data presented above was generated with optimal DNA amounts (see below and Methods section). While optimizing the 2C-ChIP protocol, we noticed that the amount of DNA used to perform the LMA step of 2C-ChIP could impact results. Indeed, using too much gDNA sometimes led to inconsistent yields (Additional file 8: Figure S2b). This is particularly important when quantitative results are desired, especially since input samples tend to be concentrated and are used for 2C-ChIP normalization (see Methods section). To determine the optimal detection range, we titrated the amount of gDNA in 2C-ChIP assays using our *HOXA* region primer set to find that linearity is observed ranging from 0.16 ng to 1.6 ng and recommend using 1–2 ng as it provides very reproducible yields as quantified by TaqMan (Additional file 8: Figure S2a, c, d). We sequenced these 2C-ChIP samples to assess how gDNA concentrations might affect the quality of libraries, and mapped the reads to our expected forward-reverse pairings. We observe that both the total read number and percentage of mapped 2C-ChIP products are maintained in this nanogram range (Additional file 8: Figure S2e), while a very low quantity of gDNA generates fewer reads and a greater percentage of non-specific ligation products between forward and reverse primers (“off-diagonal pairs”; Additional file 8: Figure S2e, f, g). Based on these results, we chose to use approximately 1.6 ng of input gDNA for each of our 2C-ChIP experiments.

### Quantitative detection of epigenomic changes at the *HOXA* cluster by 2C-ChIP during cell differentiation

**Fig. 4:**
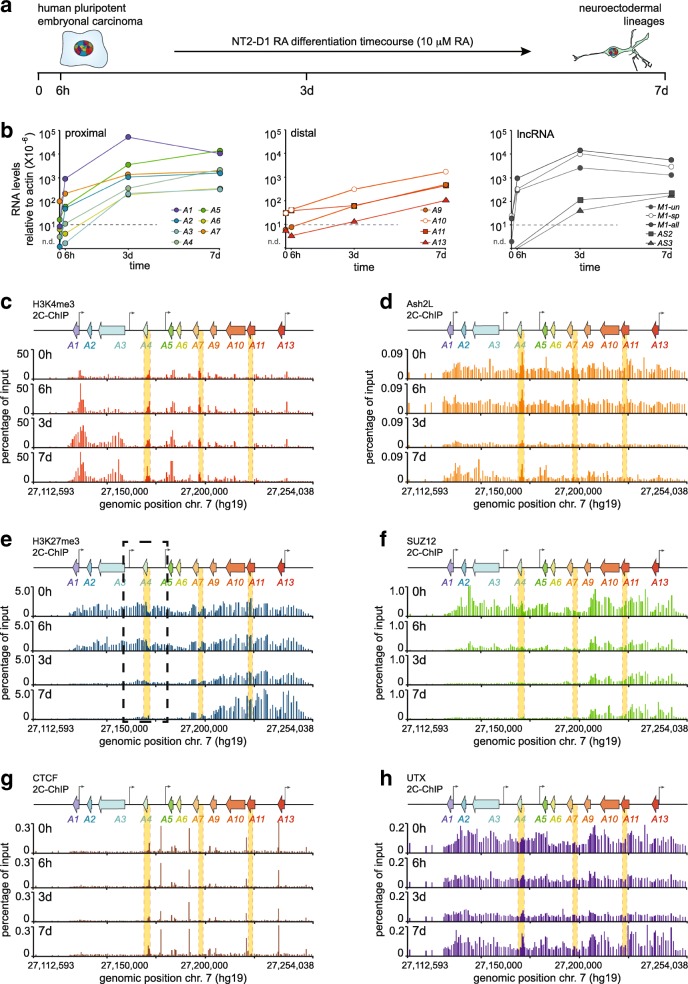
2C-ChIP analysis of a differentiation time course identifies a *HOXA* cluster region resistant to H3K27me3 demethylation. **a** Time points of RA-induced NT2-D1 differentiation examined in this study. **b** Steady state transcript levels of *HOXA* and *lncRNA* genes as measured by RT-qPCR during RA treatment. RNA levels relative to actin are shown on the *y-axis.* Dashed lines indicate values below which measurements are unreliable. Oligonucleotide sequences used for quantification are shown in Additional file 1: Table S1. All RNA measurements are from at least three PCRs. **c-h** 2C-ChIP analysis of H3K4me3 and H3K27me3 levels, and of the chromatin-binding proteins Ash2L, SUZ12, CTCF, and UTX during the RA induction time course. 2C-ChIP tracks are presented as described in Fig. 2f and Additional file 7: Figure S1. The dashed black box identifies a *HOXA* cluster region slow to lose H3K27me3 signal. 2C-ChIP BED files including surrounding negative controls are in Additional file 6: BED file 7–30

Having optimized 2C-ChIP, we then applied it to an NT2-D1 differentiation time course (Fig. 4a). Our goal was to exploit 2C-ChIP’s flexibility to analyze numerous ChIP libraries simultaneously, and explored this by probing six different chromatin components (*below*) across four time points. As done when developing and optimizing 2C-ChIP, we first measured gene expression at the *HOXA* cluster in our sample sets by RT-qPCR (Fig. 4b). We observed that treatment with RA dramatically induced the proximal *HOXA* and *lncRNA* genes, and that induction patterns in these datasets correlate well with the first datasets used to devise 2C-ChIP (Additional file 9: Figure S3). Notably, we found that gene induction proceeded in a manner that is consistent with the known collinear activation of *HOXA* genes during development. Indeed, while the most 3′ proximal genes (*HOXA1*, and *HOTAIRM1*) were highly and rapidly expressed after the addition of RA (6 h), other proximal genes were induced more progressively – not yet reaching a plateau even 7 days post-induction while *HOXA1* and *HOTAIRM1* expression had started to decline by that time. This is in contrast to distal *HOXA* transcripts, which accumulated over time in this dataset.

We tested whether expected epigenetic changes accompanied gene induction in these sample sets using RT-qPCR and found higher H3K4me3 at proximal gene promoters accompanied with decreased H3K27me3 levels specifically at induced genes over the time course (Additional file 10: Figure S4a). These changes correlated well with those measured by RT-qPCR in the first RA-induced datasets (Additional file 10: Figure S4b). We then used the fixed chromatin preparations to measure changes in the levels of H3K4me3, Ash2L, H3K27me3, SUZ12, CTCF, and UTX over the *HOXA* cluster region by 2C-ChIP (Fig. 4c-h; Additional file 6: BED file 7–30). As with our first datasets, the 2C-ChIP results obtained for H3K4me3 and H3K27me3 in the time course correlated highly with data measured by ChIP-qPCR, and with available ChIP-seq datasets from untreated NT2-D1 samples (Additional file 10: Figure S4c, d). Importantly, there was a very strong correlation between 2C-ChIP biological replicates (R^2^ ranging from 0.82 to 0.96), except for Ash2L and UTX, which upon closer inspection exhibited a high background within regions residing outside of the *HOXA* cluster serving as a negative control (Additional file 11: Figure S5; Additional file 12: Figure S6; Additional file 6: BED file 31–36).

### 2C-ChIP analysis identifies a *HOXA* cluster region slow to demethylate

From the 2C-ChIP analysis of our differentiation time course, we found that rapidly activated genes (*HOXA1* and *HOTAIRM1*) acquire higher levels of H3K4me3 at their promoters and along gene bodies compared to those at the proximal region induced later by RA (Fig. 4c). We also observed that while the PRC2 component SUZ12 is rapidly ejected from the cluster (6 h), the removal of associated silencing H3K27me3 mark occurs at a slower pace (Fig. 4e, f). The chromatin architectural protein CTCF conversely did not display notable changes when probed with our 2C-ChIP primer design, suggesting it remains stably anchored across cellular differentiation (Fig. 4g).

Analyzing 2C-ChIP data in greater details, we noticed that a region within *HOXA* seemed less prone to loss of H3K27me3, (Fig. 4e, *dashed black box*), and even SUZ12 albeit to a lesser extent. Interestingly, sequences from this region have previously been shown to repress transcription when inserted in the drosophila genome [9]. This H3K27me3 “island” overlaps perfectly with the *HOXA-AS2* lncRNA gene induced later in the time course, and after *HOTAIRM1*. These observations suggest a functional relationship between the repressive nature of this H3K27-methylated genomic sequence and the expression of *HOXA-AS2*, perhaps through combinations of chromatin structure and conformation changes (see below).

### Integrating gene expression, 2C-ChIP, and 5C data

**Fig. 5:**
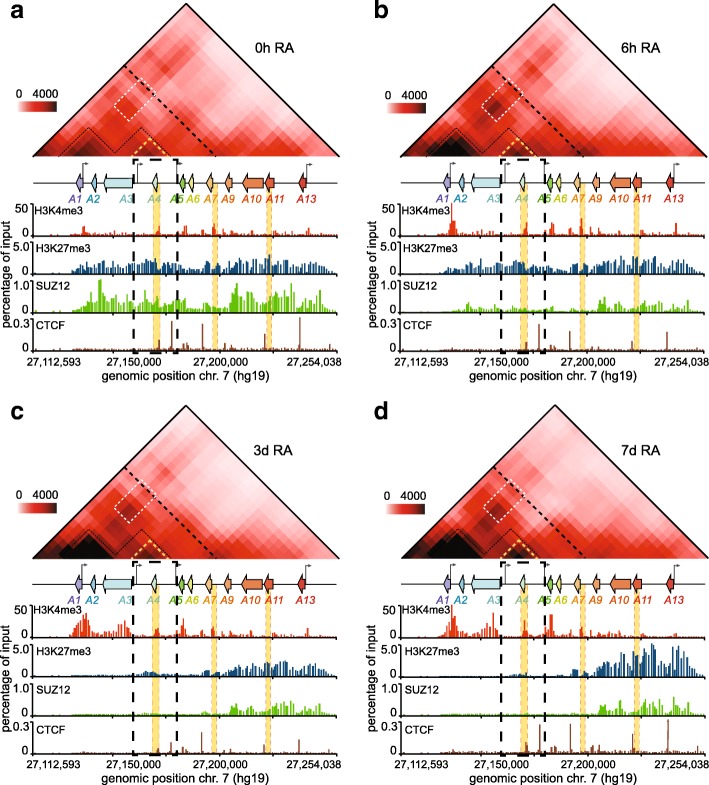
RA triggers extensive conformational changes at the *HOXA* cluster, including within a proximal subdomain where the H3K27me3 signal lingers. (**a**-**d**) The frequency of physical contacts along the *HOXA* cluster region is measured by 5C-seq in uninduced (**a**, 0 h RA; Additional file 13: 5C dataset 1), and in cells induced with RA for 6 h (**b**, 6 h RA; Additional file 13: 5C dataset 2), 3 days (**c**, 3d RA; Additional file 13: 5C dataset 3), or 7 days (**d**, 7d RA; Additional file 13: 5C dataset 4). 5C libraries were prepared with samples matched to set B. Pairwise interaction frequencies (IFs) are presented in heatmap format (10 kb bins, 10 kb smoothing, 2x steps) according to the color scale on the left of each panel. The *HOXA* cluster region is aligned to scale between each heatmap, and its corresponding 2C-ChIP tracks. The area boxed in dashed black lines within each panel highlights the region where H3K27me3 is cleared more slowly. Within heatmaps, the black dashed lines separating the proximal and distal genes indicate the TAD boundary identified previously [37]. The two main proximal subdomains found in uninduced (**a**, 0 h RA) cells are outlined in dashed black lines, and featured in the other panels as visual aids to follow conformational changes. The white boxes show long-range interactions between proximal genes. The subdomain outlined with yellow dashed lines highlights the region where H3K27 demethylation is slow. The areas shaded in yellow along the tracks identify regions characterized by ChIP-qPCR in Additional file 10: Figure S4

We then set to study how changes in H3K27me3 and other chromatin marks translate into three-dimensional chromatin organization. To accomplish this, we performed 5C-seq analysis of the *HOXA* cluster in our time course samples (Fig. 5; Additional file 13: 5C dataset 1–4; Additional file 14: Table S6). As we reported previously, RA induction changes spatial chromatin organization at the *HOXA* cluster [7]. As before, the proximal and distal parts of the cluster appeared physically separated and confined to their own topologically associating domains (TADs). Both regions were also organized into several partially overlapping subdomains (subTADs). We observed long-range contacts between early RA-induced proximal genes (*HOXA1/2*) and those destined for induction at a later time (*HOXA5/6/7*). However, in this dataset, the contact in untreated cells (0 h) was weaker than previously seen [7], and seemingly depends on the state of the actively growing population. Such variability in the strength of this long-range contact in untreated cells can lead to differences in whether or not it is captured more frequently before or after induction with RA (Additional file 15: Figure S7a). Nevertheless, we saw progressively less long-range contacts within the proximal *HOXA* region as differentiation ensued, which was accompanied by greater short-range interaction frequencies, locally where genes were most expressed (Additional file 15: Figure S7). In fact, the greatest gain of contact was within the subTAD containing *HOXA1* and *HOTAIRM1*, which are the two most induced genes of the time course. Given that the greatest increase in H3K4me3 is at this subdomain, this result suggests that here, 5C captures more contacts when genes have high H3K4me3 levels.

While the expression of some genes began tapering off seven days after RA induction (7d), we noticed that H3K4me3 continues to increase slightly over the time course, and more noticeably at *HOXA5/6/7*. This coincides with the continued induction of these genes at that time, including *HOXA-AS2*, which resides within a subdomain that progressively gains contacts during induction despite the fact that H3K27me3 continues to decrease (Fig. 5; Additional file 15: Figure S7c). Interestingly, this subdomain seemingly avoids any major interactions with neighboring genes throughout the time course (Fig. 5a-c). Considering how this area also persists in maintaining both H3K27me3 and SUZ12 after 3 days (3d), we propose that “looping out” of this region might somehow protect it from the rapid removal of PRC2. Meanwhile, at the other end of the cluster, the distal genes are kept away from the rearrangements occurring at the 3′ end – perhaps helped by the activity of CTCF bound between *HOXA6* and *HOXA7* [29] – as seen by the continuous loss of long-range contacts.

### *HOXA-AS2* as a modulator of *HOXA* cluster expression and organization

**Fig. 6:**
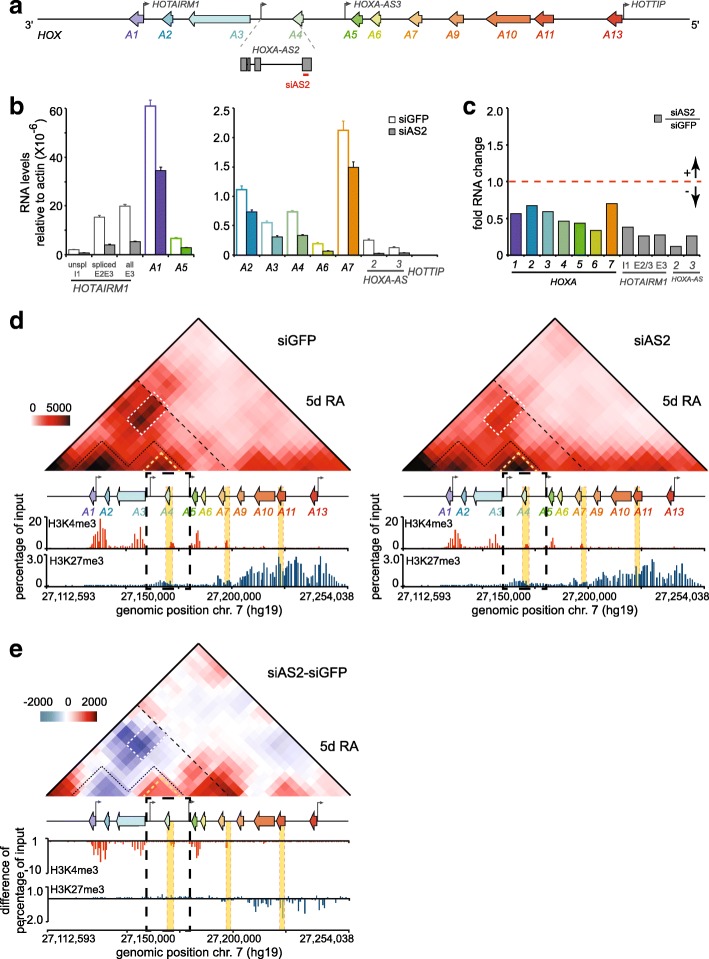
HOXA-AS2 is required to achieve high expression levels of the proximal *HOXA* genes during RA induction in NT2-D1 cells. **a** Diagram of the *HOXA* gene cluster as described in Fig. 2b. The *HOXA-AS2* gene is shown below the cluster and enlarged to better visualize the position of siAS2 used to deplete the lncRNA during RA induction (red line). **b, c** Analysis of proximal *HOXA* gene expression during RNAi knockdown of HOXA-AS2 with siAS2. Steady state levels of *HOXA* and *lncRNA* genes are measured by RT-qPCR in control (siGFP) and HOXA-AS2 (siAS2) knockdown cells induced 5 days with 10 μM RA. Fold change (**c**) is relative to the siGFP control set at 1. All measurements are from at least 3 PCRs, with error bars representing standard deviations.**d** RNAi depletion of HOXA-AS2 during RA induction leads to lower long-range contacts at the proximal *HOXA* region, and higher contact frequencies within the subdomain where it resides. Data is shown in heatmap form according to the color scale on the left and as described in Fig. 5. 2C-ChIP analysis of H3K4me3 (Additional file 6: BED file 37, 38), and H3K27me3 (Additional file 6: BED file 39, 40) in control and HOXA-AS2-knockdown cells is shown below the heatmaps. **e** Changes in the frequency of chromatin contacts occurring upon RNAi depletion of HOXA-AS2 during a 5-day RA induction. Heatmap values are IF differences between the HOXA-AS2 and control knockdown samples that are color-coded according to the scale on the left, with blue indicating a loss of contact and red showing a gain. Tracks under the differential heatmap are changes in chromatin mark levels or bound proteins detected with 2C-ChIP. Regions highlighted in heatmaps are as described in Fig. 5. The dashed black box identifies a *HOXA* cluster region slow to lose H3K27me3 signal

We wondered what role – if any – might the *HOXA-AS2* lncRNA play during *HOXA* gene induction by RA. We probed this question by examining gene expression, chromatin landscape, and three-dimensional chromatin organization of the *HOXA* cluster region in RA-induced samples depleted of the lncRNA (Fig. 6). RNAi knockdown using an siRNA against the transcript’s 3′ end considerably reduced the accumulation of HOXA-AS2 during a 5-day RA treatment. We found that this drastically reduces the expression of all the proximal *HOXA* genes (Fig. 6a, b, c). 5C-seq analysis of matching knockdown samples revealed that curbing HOXA-AS2 accumulation led to greater contact frequencies within its own subTAD, and much fewer long-range interactions within the proximal region, perhaps contributing to the lower *HOXA* levels by distancing enhancers from genes (Fig. 6d, e, *top*; Additional file 13: 5C dataset 5, 6). Moreover, 2C-ChIP analysis shows that lower H3K4me3 levels at all promoters accompanied these changes without H3K27me3 changes at gene bodies (Fig. 6d, e, *bottom*; Additional file 6: BED file 37–40). Together, these results show that HOXA-AS2 is required to enhance expression of all the cluster’s RA-responsive genes by promoting high H3K4me3 levels at promoters, perhaps by controlling the cluster’s three-dimensional chromatin organization.

## Discussion

As shown above, 2C-ChIP is a very reproducible approach to quantitatively measure ChIP signals over large, genome-scale DNA regions. It faithfully recapitulates data that would otherwise need to be generated by ChIP-qPCR or ChIP-seq. Like ChIP-qPCR, this method is quantitative but significantly less tedious. While we found that the amount of genomic DNA used to perform the LMA step of 2C-ChIP can affect the linearity of detection, this consideration is most relevant to input samples given that the amount of ChIP material generally falls within the linear detection range of the assay (Additional file 8: Figure S2b).

2C-ChIP can be used to generate multiple profiles simultaneously over defined genomic regions, and because it uses much less ChIP material per reaction it can provide much more information per sample. 2C-ChIP also permits a better assessment of the quality of immunoprecipitations compared to ChIP-qPCR because it generates more detailed ChIP profiles. Unlike other dedicated ChIP readouts such as bead-capture [31, 32], or ChIP-on-chip [33, 34], the experimental design for 2C-ChIP can be easily and rapidly changed at very little cost simply by ordering new sets of 2C-ChIP primers. The use of oligonucleotides for LMA allows for a variety of probe designs to fit most experimental needs. Probes can be designed at gene promoters, suspected DNA enhancer regions, or can be tiled at variable densities across one or more genomic loci. Also, 2C-ChIP libraries can be multiplexed to explore numerous chromatin features such as histone marks and chromatin-bound proteins simultaneously. High coverage can be achieved by limiting the size of primers pools and/or the number of sequencing targets.

High-cost modifications like biotinylation are not required and reverse 2C-ChIP primers can easily be 5′ phosphorylated in-house with T4 polynucleotide kinase to save on cost. New regions can be added and problematic primers excluded from one experiment to the next such that optimization can be quickly accomplished. Different high-throughput sequencing platforms can be selected simply by altering the two PCR amplification primers.

## Conclusions

We developed 2C-ChIP to fill the need for a versatile, low cost, high-throughput method to quantitatively measure the abundance of DNA sequences in ChIP samples. The technique combines ChIP with LMA and deep sequencing to yield a robust molecular biology tool designed to probe large genomic regions of interest. While the flexible nature of 2C-ChIP makes it an interesting tool to investigate chromatin landscape, the ever-decreasing cost of deep sequencing will continue to establish it as the most cost-effective approach for the functional analysis of chromatin.

## Methods

### Cell culture

The NTERA-2 clone D1 (NT2-D1) cells are from the American Type Culture Collection (ATCC; CRL-1973), and are cultured in Dulbecco’s Modified Eagle’s Medium (DMEM; Gibco) supplemented with 10% fetal bovine serum (FBS; Thermo Fisher Scientific) and 0.01% Penicillin/Streptomycin (‘complete’ DMEM) as described previously [27]. Gene induction is with 10 μM all-trans retinoic acid (RA; Sigma-Aldrich) as outlined in [7] . For the 3-day induced samples, the induction media is changed after 48 h with cells collected on the following day. When cells are induced for seven days, the media is changed after 48 h, the cells are passaged the next day in fresh induction media, and the induction media is changed again on the following day (96 h).

### RNA interference

HOXA-AS2 knockdown is performed by reverse transfection with the Lipofectamine® RNAiMAX reagent and 45 nM of small interfering RNA (siRNA) as instructed by the manufacturer (Thermo Fisher Scientific). The control siRNA (siGFP; 5′-GCAAGCTGACCCTGAAGTTC-3′) is from GE Healthcare Dharmacon Inc. (cat. no. P-002048-01-20). HOXA-AS2 siRNAs (siAS2; 5′-CAAGCUAUCUACAAGGUUUTT-3′) are from Ambion® (Silencer® Select siRNAs). To induce cells, complete DMEM containing 10 μM RA is added 6 h after transfection. The cells are passaged and re-transfected in RA-containing media after 48 h as described above and collected at the 5-day induction time point.

### RNA extraction and quantitative real-time polymerase chain reaction (RT-qPCR)

Total RNA is extracted with the TRIzol® reagent (Thermo Fisher Scientific) as per the manufacturer’s instructions. The RNA is then treated with DNAseI (RNase-free; New England BioLabs Inc.) for 15 min at 37 °C, and re-extracted with TRIzol®. For gene expression analysis, 1 μg of DNAsed total RNA is used to generate cDNA with the SuperScript™ III reverse transcriptase and oligo(dT)_20_ (Thermo Fisher Scientific). Quantitative real-time polymerase chain reaction (RT-qPCR) is performed as described previously [10]. The primer sequences used for this analysis are presented in Additional file 1: Table S1.

### Chromatin immunoprecipitation (ChIP)

ChIP experiments are conducted as detailed previously [10] with cells fixed in 1% formaldehyde for 10 min and quenched with 0.125 M glycine. Each ChIP is performed with 5 million cells, and 5 μg of one of the following antibodies: H3K4me3 (Abcam; ab8580), H3K27me3 (Abcam; ab6002), SUZ12 (Abcam; ab12073), UTX (Bethyl Laboratories; A302-374A), Ash2L (Bethyl Laboratories; A300-489A), CTCF (Millipore; 07–729), or control IgG (Abcam; ab37415). Magnetic Dynabeads® Protein G are used for pull down according to manufacturer’s instructions (Thermo Fisher Scientific). 10% of input chromatin is set aside and the purified genomic DNA (gDNA) is used as normalization control (see below). Final input and ChIP DNA are re-suspended in 50 μl of 1X Tris-EDTA pH 8.

### Carbon copy chromatin immunoprecipitation (2C-ChIP)

The goal of this study was to develop a high-throughout methodology that can be used to profile specific genomic regions by ChIP. We designed this new approach to be quantitative, simple to implement, versatile in its design, and inexpensive. Below, we outline experimental details that complement our ‘Results and discussion’ section and that are essential to optimally implement the technique.

### 2C-ChIP primer design

Forward and reverse primers were designed complementary to the antisense strand in the human genome reference (hg19). The primer sequences were selected to have a GC content ranging between ~ 40–60%, unique BLAT results, and limited possibilities in self-annealing and/or hairpin formation as estimated by the ‘Oligo Calc’ bioinformatics tool available at: http://biotools.nubic.northwestern.edu/OligoCalc.html. Primer homology lengths vary between 22 and 34 nucleotides. Forward 2C-ChIP primers include a 5′-end tail corresponding to the complementary sequence of a modified T3 Universal primer (5′-TAATTGGGAGTGATTTCCCT-3′) (Fig. 1b, *top*). Reverse 2C-ChIP primers are designed to include a 3′-end tail corresponding to the complementary P1-key sequence (5′-ATCACCGACTGCCCATAGAGAGG-3′) used with the PGM™ sequencing system (Thermo Fisher Scientific). The 2C-ChIP primer sequences used in this study are presented in Additional file 4 Table S4.

### 2C-ChIP library preparation

To generate 2C-ChIP libraries, purified gDNA from ChIP samples (1% of H3K4me3 or H3K27me3 ChIPs; 10% of Ash2L, SUZ12, UTX, or CTCF; from 5 million cells and 5 μg antibody as described above), or 1 to 2 ng of the corresponding input gDNA is mixed with 1.5 μg of salmon testis DNA (Sigma-Aldrich; cat. no. D7656), and 0.34 fmol of each 2C-ChIP primer (160 forward-reverse primer pairs in this study; final primer concentration of 34 pM), in annealing buffer (1X NEBuffer™ 4; New England Biolabs; cat. no. B7004S) and a final reaction volume of 10 μl. The amount of input gDNA is estimated using the Quant-iT™ PicoGreen™ dsDNA Assay Kit as instructed by the manufacturer (Thermo Fisher Scientific; cat. no. P11496).

Samples are denatured at 95 °C for 5 min and annealed overnight at 55 °C. Annealed primer pairs are ligated the next day for 1 h at 55 °C with 10 units of Taq DNA ligase (New England Biolabs; cat. no. M0208S) by adding 20 μl of ligation buffer (25 mM Tris-HCl at pH 7.6, 31.25 mM potassium acetate, 12.5 mM magnesium acetate, 1.25 mM NAD, 12.5 mM DTT, 0.125% Triton X-100) containing the enzyme. The reactions are terminated by heat-inactivating the enzyme at 65 °C for 15 min.

2C-ChIP libraries (14 μl) are then PCR-amplified 16 cycles in a total reaction volume of 100 μl using the ‘2C-ChIP library amplification primers’ designed for sequencing on the PGM™ system (Fig. 1b, *bottom*). These amplification primers can be modified as desired to be compatible with any other chosen sequencing platform. Here, the forward primer consists of (5′-3′): a forward A-key sequence followed by a barcode, then a modified T3 sequence. The reverse primer simply corresponds to the Ion Torrent™ P1-key sequence that will hybridize onto the sequencing chips.

PCR amplicons of ~ 160 bp are next size-selected from the reactions using Sera-Mag SpeedBeads™ (Thermo Fisher Scientific; cat. no. 09–981-123). Purified libraries are then quantified using the Ion Library TaqMan™ Quantitation Kit (Thermo Fisher Scientific; cat. no. 4468802) to estimate the proper dilution for sequencing. 2C-ChIP libraries are multiplexed (up to 10 libraries per sequencing run) at a combined 9 pM concentration, and processed for sequencing with a PGM™ system (Thermo Fisher Scientific) as previously described [8] using the Ion 316™ chip kit v2 (Thermo Fisher Scientific; cat. no. 4483324).

### 2C-ChIP paired-end read handling

The FASTQ sequencing files of the multiplexed 2C-ChIP runs are first converted to a FASTA format and mapped to a custom reference sequence assembly featuring all possible barcode-Forward and Reverse 2C-ChIP primer combinations. Mapping is performed using BLAST (v2.5.0; [35]) with a minimum word size of 50, corresponding to the smallest expected product size. Reads not uniquely mapped after this iteration are re-mapped to a second custom reference sequence assembly consisting of the unpaired barcode-Forward, Forward, or Reverse 2C-ChIP primer sequences, using a minimum word size of 22 that corresponds to the length of one Forward primer – again, the smallest expected product size.

This two-step mapping approach reduces the search space and runtime of the BLAST algorithm on this type of data because a majority of paired-end reads will align to the first database while short ones (i.e. those shorter than the minimum word length) will not uniquely map. The 2C-ChIP data from individual samples is next divided by barcodes. Each dataset is then normalized to quantitatively express the 2C-ChIP data relative to input. First, all datasets are normalized for read count. Next, all datasets are adjusted for the dilution made for sequencing based on TaqMan™ quantitation. Data from the ChIP samples and their corresponding inputs is also adjusted to account for dilutions made to ensure linear 2C-ChIP detection. The ChIP sample data is further adjusted to account for the fact that only 10% of the input is processed. Finally, the ratio of normalized ChIP to normalized input is calculated.

### Chromosome conformation capture carbon copy (5C)

#### 5C library preparation

3C libraries are generated from aliquots of 2 million cells fixed in 1% formaldehyde for 10 min and quenched with 0.125 M glycine. The 3C libraries are made using the *Bgl*II restriction enzyme as we described previously [36]. The 5C libraries are generated using the alternating Forward/Reverse (FR) 5C primers listed in Additional file 14: Table S6, and are sequenced as published previously [30].

#### 5C paired-end read handling

5C paired-end reads are sequenced on a PGM™ system (Thermo Fisher Scientific) as previously described [8] using the Ion 314™ chip kit v2 BC (Thermo Fisher Scientific; cat. no. 4488144). Sequenced products are mapped to a custom reference sequence assembly featuring all expected ligation products between Forward and Reverse 5C primers using the tools described in [8]. Mapped products were normalized for read count (reads per million) for comparison between libraries.

#### Databases and URLs

The 2C-ChIP sequencing data generated for this study can be downloaded from the Sequence Read Archive (SRA) website with the following link: https://www.ncbi.nlm.nih.gov/sra/SRP155022; SRA accession number SRP155022. The NT2-D1 ChIP-seq data for H3K4me3 (wgEncodeEH000909), H3K27me3 (wgEncodeEH000908), and SUZ12 (wgEncodeEH000652) can be downloaded and visualized at the Encyclopedia of DNA Elements at UCSC (http://www.epigenomebrowser.org/ENCODE/). The ‘my5C-heatmap’ bioinformatics tool can be found at the 3DG browser (my5C; http://3DG.umassmed.edu).

## Additional files

Additional file 1: **Table S1.** *HOXA* and *lncRNA* gene expression in NT2-D1 cells before and after a 3-day treatment with retinoic acid (RA; 10 micromolar) as measured by RT-qPCR. This table contains steady-state mRNA quantifications from at least 3 independent PCRs in two biological replicates. (XLSX 14 kb)

Additional file 2: **Table S2.** List of primer sequences used to quantify gene expression by quantitative real-time polymerase chain reaction (RT-qPCR). (XLSX 11 kb)

Additional file 3: **Table S3.** List of primer sequences used to PCR-quantify DNA regions isolated by chromatin immunoprecipitation (ChIP-qPCR). (XLSX 10 kb)

Additional file 4: **Table S4.** List of 2C-ChIP primer sequences used in this study. (XLSX 70 kb)

Additional file 5: **Table S5.** List of primer sequences used to amplify 2C-ChIP libraries prior to deep sequencing on a PGM™ system (Thermo Fisher Scientific). (XLSX 54 kb)

Additional file 6: **BED files.** This folder contains the input-normalized 2C-ChIP data in BedGraph format of all the ChIP samples measured in this study. **BED files 1 and 2:** H3K4me3 in untreated (0h; **1**) and RA-treated (3d; **2**) NT2-D1 (setA). **BED files 3 and 4:** H3K27me3 in untreated (0h; **3**) and RA-treated (3d; **4**) NT2-D1 (setA). **BED files 5 and 6:** SUZ12 in untreated (0h; **5**) and RA-treated (3d; **6**) NT2-D1 (setA). **BED files 7, 8, 9, and 10:** H3K4me3 in untreated (0h; **7**) and RA-treated (6h; **8**, 3d; **9**, 7d; **10**) NT2-D1 (setB). **BED files 11, 12, 13, and 14:** Ash2L in untreated (0h; **11**) and RA-treated (6h; **12**, 3d; **13**, 7d; **14**) NT2-D1 (setB). **BED files 15, 16, 17, and 18:** H3K27me3 in untreated (0h; **15**) and RA-treated (6h; **16**, 3d; **17**, 7d; **18**) NT2-D1 (setB). **BED files 19, 20, 21, and 22:** SUZ12 in untreated (0h; **19**) and RA-treated (6h; **20**, 3d; **21**, 7d; **22**) NT2-D1 (setB). **BED files 23, 24, 25, and 26:** CTCF in untreated (0h; **23**) and RA-treated (6h; **24**, 3d; **25**, 7d; **26**) NT2-D1 (setB). **BED files 27, 28, 29, and 30:** UTX in untreated (0h; **27**) and RA-treated (6h; **28**, 3d; **29**, 7d; **30**) NT2-D1 (setB). **BED files 31 and 32:** Ash2L in untreated (0h; **31**) and RA-treated (3d; **32**) NT2-D1 (setA). **BED files 33 and 34:** CTCF in untreated (0h; **33**) and RA-treated (3d; **34**) NT2-D1 (setA). **BED files 35 and 36:** UTX in untreated (0h; **35**) and RA-treated (3d; **36**) NT2-D1 (setA). **BED files 37 and 38:** H3K4me3 in control (siGFP; **37**) or knockdown (siAS2; **38**) RA-induced (5d) NT2-D1. **BED files 39 and 40:** H3K27me3 in control (siGFP; **39**) or knockdown (siAS2; **40**) RA-induced (5d) NT2-D1. (CPGZ 69 kb)

Additional file 7: **Figure S1.** SUZ12 binding analysis by 2C-ChIP and ChIP-qPCR correlate well at the *HOXA* cluster. a 2C-ChIP analysis of SUZ12 at the *HOXA* gene cluster before and upon RA treatment for 3 days. Data shown is limited to the gene-encoding region and excludes most of the surrounding negative controls. Complete BED files are in Additional file 6: BED file 5, 6. Primer sequences are found in Additional file 4: Table S4. b ChIP-qPCR analysis of SUZ12 at select *HOXA* genes upon a 3-day RA treatment. Primer sequences are shown in Additional file 3: Table S3, and regions probed are highlighted in yellow in panel a. Error bars are standard deviations from at least 3 PCRs. c Correlation between ChIP-qPCR results and corresponding 2C-ChIP signals for the SUZ12 ChIP in uninduced and 3-day RA-induced NT2-D1 cells (Spearman’s rho = 0.81). (PDF 373 kb)

Additional file 8: **Figure S2.** Defining the optimal 2C-ChIP linear detection range. **a** Diagram of the HOXA cluster region probed by 2C-ChIP. Numbers above indicate the position on chromosome 7 (hg19). Color-coded arrows represent protein-coding genes. Grew arrows indicate the transcription start site (TSS) of lncRNAs. The position of 2C-ChIP primer pairs (160) is shown below the genomic region. **b** Using high levels of genomic DNA (gDNA) in 2C-ChIP can yield variable product concentrations. Three libraries (technical replicate 1, 2, 3) were generated using the 2C-ChIP primers (**a**), and 16 ng of input gDNA. Multiple volumes of the resulting 2C-ChIP samples were quantified by TaqMan to illustrate how high gDNA levels can affect results. Estimated TaqMan concentrations are indicated on the top right of each graph. **c** Titrating the optimal range of gDNA amount to produce 2C-ChIP samples. Dilution scheme of the input gDNA used to generate 2C-ChIP libraries quantified in **d** by TaqMan. **d** 2C-ChIP libraries were produced from two independent input gDNA sources (biological replicates; biol. rep. 1, 2) to assess 2C-ChIP reproducibility. **e** Using low gDNA amounts in 2C-ChIP leads to lower quality sequencing runs. The 2C-ChIP libraries quantified in **d** were sequenced on a PGMTM system to show that both total reads and percentage of expected mappable pairs decrease when very low gDNA amounts are used to generate 2C-ChIP samples. Expected mappable pairs are those between adjacent forward and reverse primers. **f**, **g** Low gDNA amount in 2C-ChIP assays increases the incidence of non-specific ligation between 2C-ChIP primers. Most unexpected sequence reads (~92%) consist of products between non-adjacent (off-diagonal) primer pairs. The optimal 2C-ChIP linear detection range highlighted in orange (panels **c**, **d**, and **g**) is based both on reproducible yield and high percentage of expected mappable reads. (PDF 469 kb)

Additional file 9: **Figure S3.** The basal and 3-day induced gene expression levels from two biological replicates correlate well with each other. Scatter plot analysis of steady state transcript levels measured by RT-qPCR before (0 h) and after RA induction (3d). ‘Set A’ measurements are those from the first induction set presented in Fig. 2, used to develop and optimize 2C-ChIP. ‘Set B’ data is from the differentiation time course. (PDF 340 kb)

Additional file 10: **Figure S4.** ChIP-qPCR and 2C-ChIP analysis of the RA-induced differentiation time course (dataset B) correlates well with the first induction dataset (dataset A), and with ChIP-seq results. a ChIP-qPCR analysis of the H3K4me3 and H3K27me3 level changes at select *HOXA* genes during the time course. Primer sequences are shown in Additional file 3: Table S3, and regions probed are highlighted in yellow in Fig. 4c, *E. error* bars are standard deviations from at least 3 PCRs. b Scatter plot correlation between ChIP-qPCR results from the two biological replicates (‘set A’ and ‘set B’). c Spearman correlation between ChIP-qPCR and corresponding 2C-ChIP signals for H3K4me3 and H3K27me3 ChIPs during the time course (Spearman’s rho = 0.88 and 0.94, respectively). d 2C-ChIP data from set B and ChIP-seq results correlate well at the *HOXA* gene cluster. 2C-ChIP analysis of H3K4me3 (left), H3K27me3 (middle), and SUZ12 (right) in uninduced NT2-D1 cells from set B display a high degree of similarity with ChIP-seq data despite the fact that different antibodies were used, and that ChIP samples were prepared by different labs. 2C-ChIP and ChIP-seq correlations are between regions featured in both assays and exclude measurements equal to zero. Spearman’s rho is indicated on the bottom right of each graph. Compared 2C-ChIP datasets are those from Additional file 6: BED file 7, 15, 19. (PDF 535 kb)

Additional file 11: **Figure S5.** Comparison of two biological replicates shows that 2C-ChIP is highly reproducible. The uninduced (0 h) and RA-induced (3d) 2C-ChIP data from set A and B NT2-D1 cells are highly correlated except for Ash2L and UTX, which display high background levels in negative control regions (Additional file 6: BED file 11, 13, 27, 29). (PDF 434 kb)

Additional file 12: **Figure S6.** 2C-ChIP results for Ash2L, CTCF, and UTX in set A. Data is displayed as outlined in Fig. 2f. The complete BED files including surrounding negative controls are in Additional file 6: BED file 31–36). (PDF 377 kb)

Additional file 13: 5C datasets. This folder contains the read count-normalized 5C data in matrix format (.txt) of all the 5C samples produced for this study. These files can be uploaded directly to the 3DG browser (my5C; http://3DG.umassmed.edu). 5C dataset 1. 5C analysis of the *HOXA* cluster region in untreated (0 h) NT2-D1 (set B). 5C dataset 2. 5C analysis of the *HOXA* cluster region in RA-treated (6 h) NT2-D1 (set B). 5C dataset 3. 5C analysis of the *HOXA* cluster region in RA-treated (3d) NT2-D1 (set B). 5C dataset 4. 5C analysis of the *HOXA* cluster region in RA-treated (7d) NT2-D1 (set B). 5C dataset 5. 5C analysis of the *HOXA* cluster region in control (siGFP) NT2-D1 cells induced with RA for 5 days. 5C dataset 6. 5C analysis of the *HOXA* cluster region in test (siAS2) NT2-D1 cells induced with RA for 5 days. (CPGZ 22 kb)

Additional file 14: **Table S6.** List of primer sequences used for chromatin conformation capture carbon copy (5C). (XLSX 10 kb)

Additional file 15: **Figure S7.** RA-induced NT2-D1 differentiation is accompanied by extensive conformational and epigenomic changes along the *HOXA* gene cluster. Changes in the frequency of chromatin contacts occurring early after RA induction (a; 0 – 6 h RA), after 3 days (b; 6 h – 3d RA), or in the later phase of the time course (c; 3d – 7d) are shown in heatmap form. Heatmap values represent IF differences between later and earlier time points that are color-coded according to the scale in panel a, with blue indicating a loss of contact and red an interaction gain. Regions highlighted in heatmaps are as described in Fig. 5. Tracks under each heatmap represent corresponding changes in the levels of chromatin marks or bound proteins detected with 2C-ChIP. The dashed black box identifies a *HOXA* cluster region slow to lose H3K27me3 signal (PDF 572 kb)

## Abbreviations

3C: Chromosome conformation capture
5C: Chromatin conformation capture carbon copy
ChIP: Chromatin immunoprecipitation
RNAi: RNA interference
TSS: Transcriptional start site
