# Supplementary figures and images for: 2C-ChIP: measuring chromatin immunoprecipitation signal from defined genomic regions with deep sequencing
Source: BMC Genomics. 2019 Feb 28;20:162. doi: 10.1186/s12864-019-5532-5 (PMC6394006; doi:10.1186/s12864-019-5532-5)

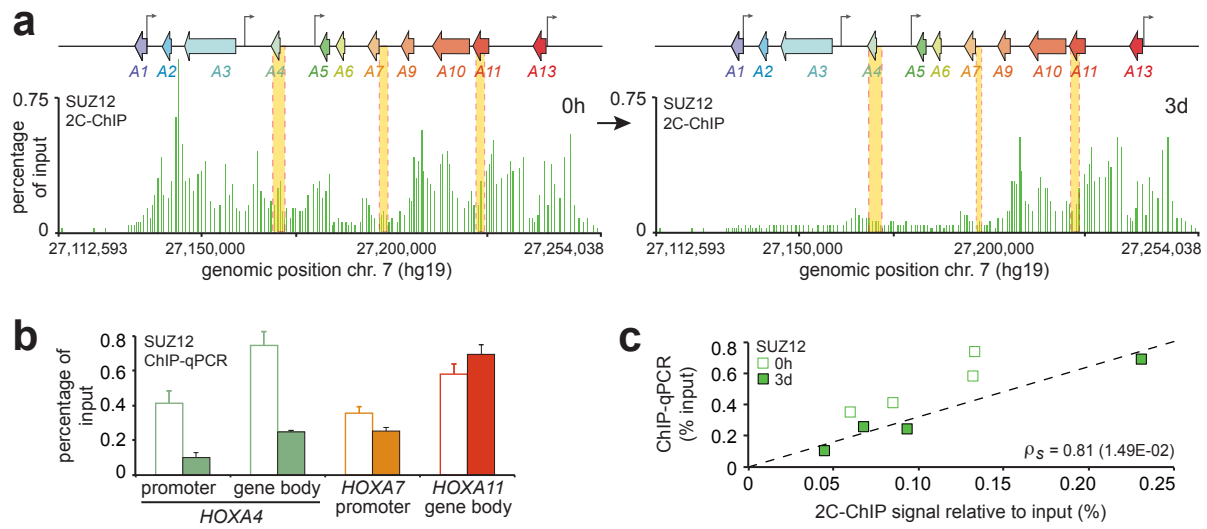

Supplement: Supplementary file 7 — Figure S1. SUZ12 binding analysis by 2C-ChIP and ChIP-qPCR correlate well at the HOXA cluster. a 2C-ChIP analysis of SUZ12 at the HOXA gene cluster before and upon RA treatment for 3 days. Data shown is limited to the gene-encoding region and excludes most of the surrounding negative controls. Complete BED files are in Additional file 6: BED file 5, 6. Primer sequences are found in Additional file 4: Table S4. b ChIP-qPCR analysis of SUZ12 at select HOXA genes upon a 3-day RA treatment. Primer sequences are shown in Additional file 3: Table S3, and regions probed are highlighted in yellow in panel a. Error bars are standard deviations from at least 3 PCRs. c Correlation between ChIP-qPCR results and corresponding 2C-ChIP signals for the SUZ12 ChIP in uninduced and 3-day RA-induced NT2-D1 cells (Spearman’s rho = 0.81). (PDF 373 kb) [file 12864_2019_5532_MOESM7_ESM.pdf]

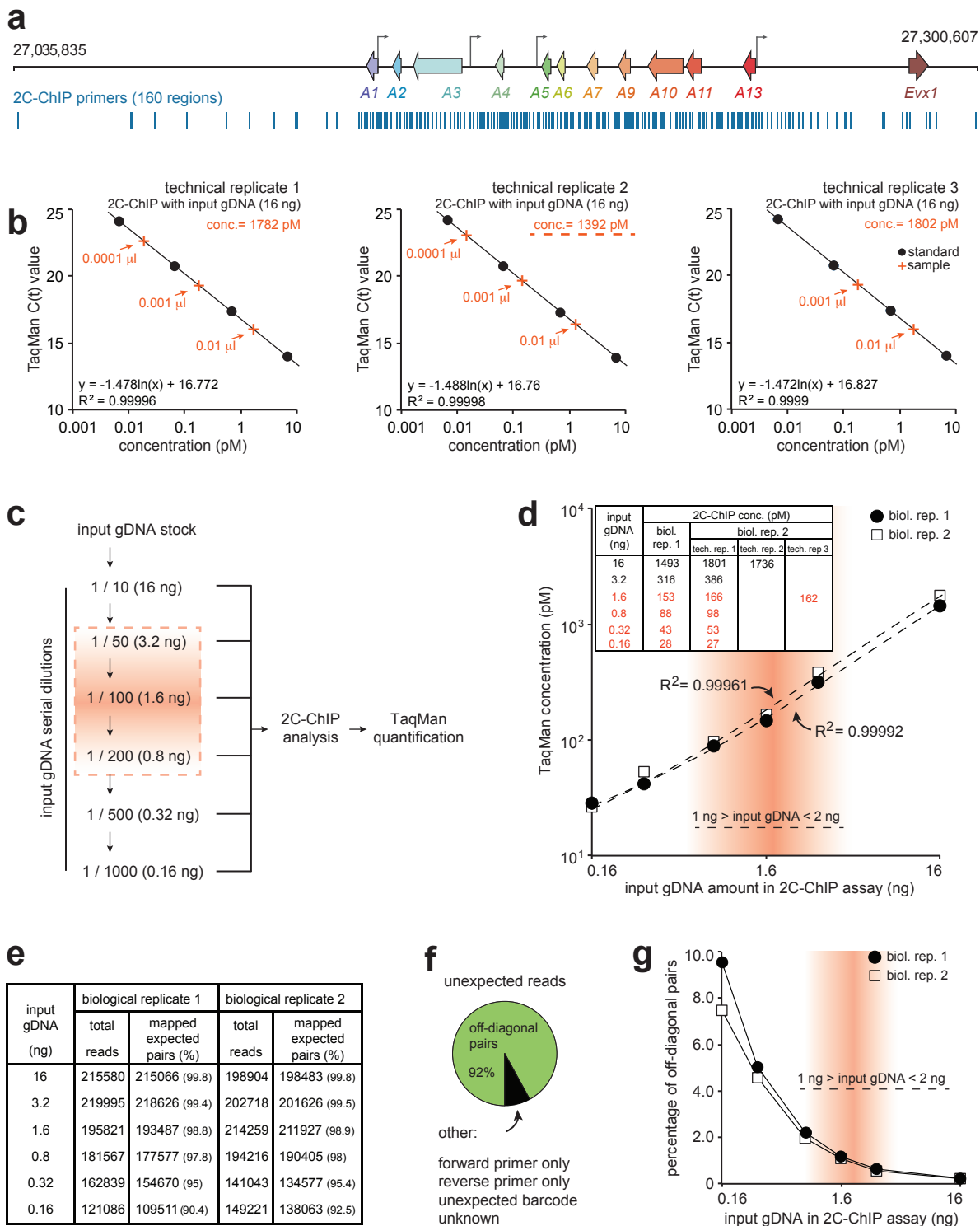

Supplement: Supplementary file 8 — Figure S2. Defining the optimal 2C-ChIP linear detection range. a Diagram of the HOXA cluster region probed by 2C-ChIP. Numbers above indicate the position on chromosome 7 (hg19). Color-coded arrows represent protein-coding genes. Grew arrows indicate the transcription start site (TSS) of lncRNAs. The position of 2C-ChIP primer pairs (160) is shown below the genomic region. b Using high levels of genomic DNA (gDNA) in 2C-ChIP can yield variable product concentrations. Three libraries (technical replicate 1, 2, 3) were generated using the 2C-ChIP primers (a), and 16 ng of input gDNA. Multiple volumes of the resulting 2C-ChIP samples were quantified by TaqMan to illustrate how high gDNA levels can affect results. Estimated TaqMan concentrations are indicated on the top right of each graph. c Titrating the optimal range of gDNA amount to produce 2C-ChIP samples. Dilution scheme of the input gDNA used to generate 2C-ChIP libraries quantified in d by TaqMan. d 2C-ChIP libraries were produced from two independent input gDNA sources (biological replicates; biol. rep. 1, 2) to assess 2C-ChIP reproducibility. e Using low gDNA amounts in 2C-ChIP leads to lower quality sequencing runs. The 2C-ChIP libraries quantified in d were sequenced on a PGMTM system to show that both total reads and percentage of expected mappable pairs decrease when very low gDNA amounts are used to generate 2C-ChIP samples. Expected mappable pairs are those between adjacent forward and reverse primers. f, g Low gDNA amount in 2C-ChIP assays increases the incidence of non-specific ligation between 2C-ChIP primers. Most unexpected sequence reads (~92%) consist of products between non-adjacent (off-diagonal) primer pairs. The optimal 2C-ChIP linear detection range highlighted in orange (panels c, d, and g) is based both on reproducible yield and high percentage of expected mappable reads. (PDF 469 kb) [file 12864_2019_5532_MOESM8_ESM.pdf]

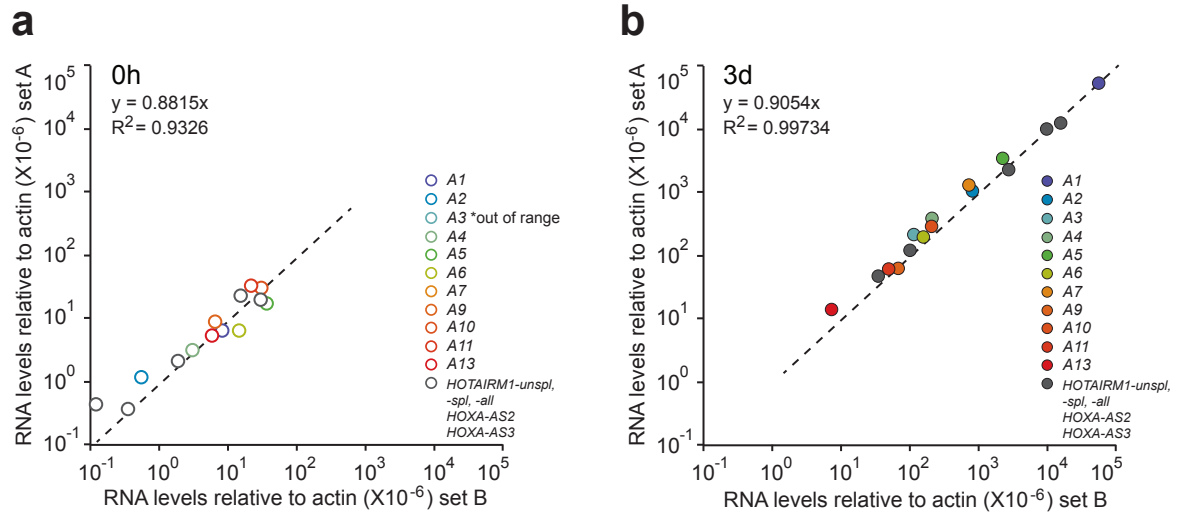

Supplement: Supplementary file 9 — Figure S3. The basal and 3-day induced gene expression levels from two biological replicates correlate well with each other. Scatter plot analysis of steady state transcript levels measured by RT-qPCR before (0 h) and after RA induction (3d). ‘Set A’ measurements are those from the first induction set presented in Fig. 2, used to develop and optimize 2C-ChIP. ‘Set B’ data is from the differentiation time course. (PDF 340 kb) [file 12864_2019_5532_MOESM9_ESM.pdf]

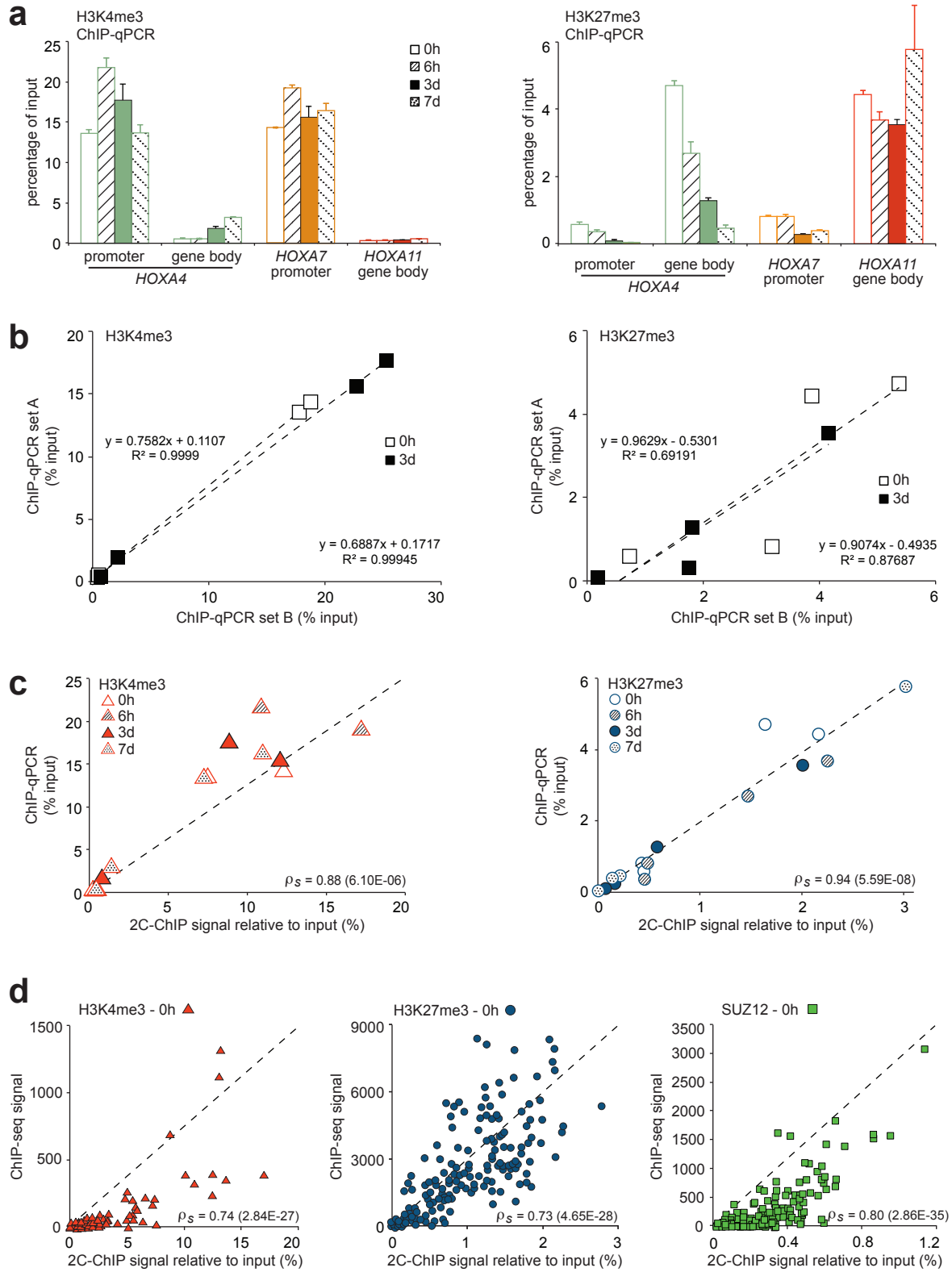

Supplement: Supplementary file 10 — Figure S4. ChIP-qPCR and 2C-ChIP analysis of the RA-induced differentiation time course (dataset B) correlates well with the first induction dataset (dataset A), and with ChIP-seq results. a ChIP-qPCR analysis of the H3K4me3 and H3K27me3 level changes at select HOXA genes during the time course. Primer sequences are shown in Additional file 3: Table S3, and regions probed are highlighted in yellow in Fig. 4c, E. error bars are standard deviations from at least 3 PCRs. b Scatter plot correlation between ChIP-qPCR results from the two biological replicates (‘set A’ and ‘set B’). c Spearman correlation between ChIP-qPCR and corresponding 2C-ChIP signals for H3K4me3 and H3K27me3 ChIPs during the time course (Spearman’s rho = 0.88 and 0.94, respectively). d 2C-ChIP data from set B and ChIP-seq results correlate well at the HOXA gene cluster. 2C-ChIP analysis of H3K4me3 (left), H3K27me3 (middle), and SUZ12 (right) in uninduced NT2-D1 cells from set B display a high degree of similarity with ChIP-seq data despite the fact that different antibodies were used, and that ChIP samples were prepared by different labs. 2C-ChIP and ChIP-seq correlations are between regions featured in both assays and exclude measurements equal to zero. Spearman’s rho is indicated on the bottom right of each graph. Compared 2C-ChIP datasets are those from Additional file 6: BED file 7, 15, 19. (PDF 535 kb) [file 12864_2019_5532_MOESM10_ESM.pdf]

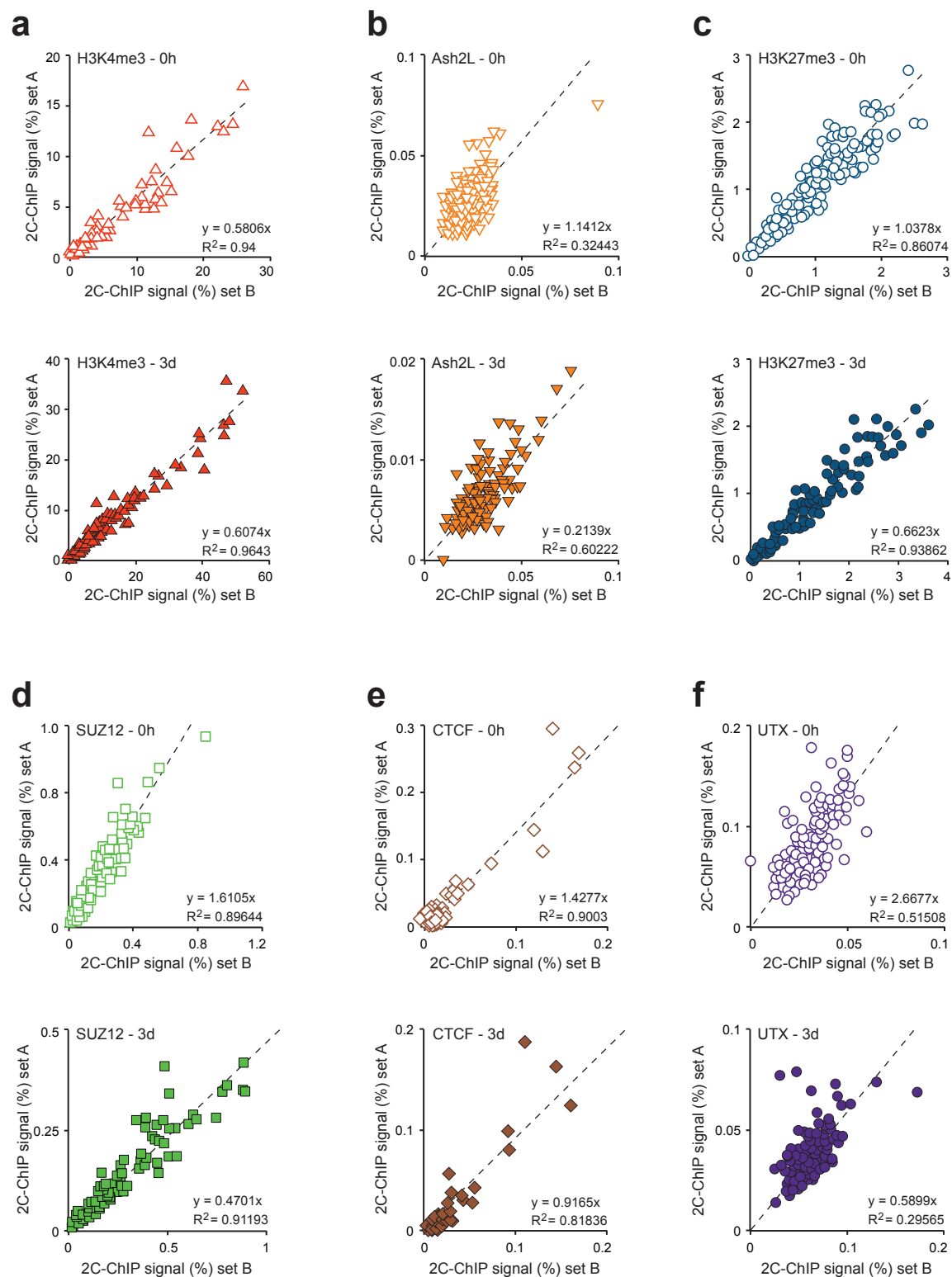

Supplement: Supplementary file 11 — Figure S5. Comparison of two biological replicates shows that 2C-ChIP is highly reproducible. The uninduced (0 h) and RA-induced (3d) 2C-ChIP data from set A and B NT2-D1 cells are highly correlated except for Ash2L and UTX, which display high background levels in negative control regions (Additional file 6: BED file 11, 13, 27, 29). (PDF 434 kb) [file 12864_2019_5532_MOESM11_ESM.pdf]

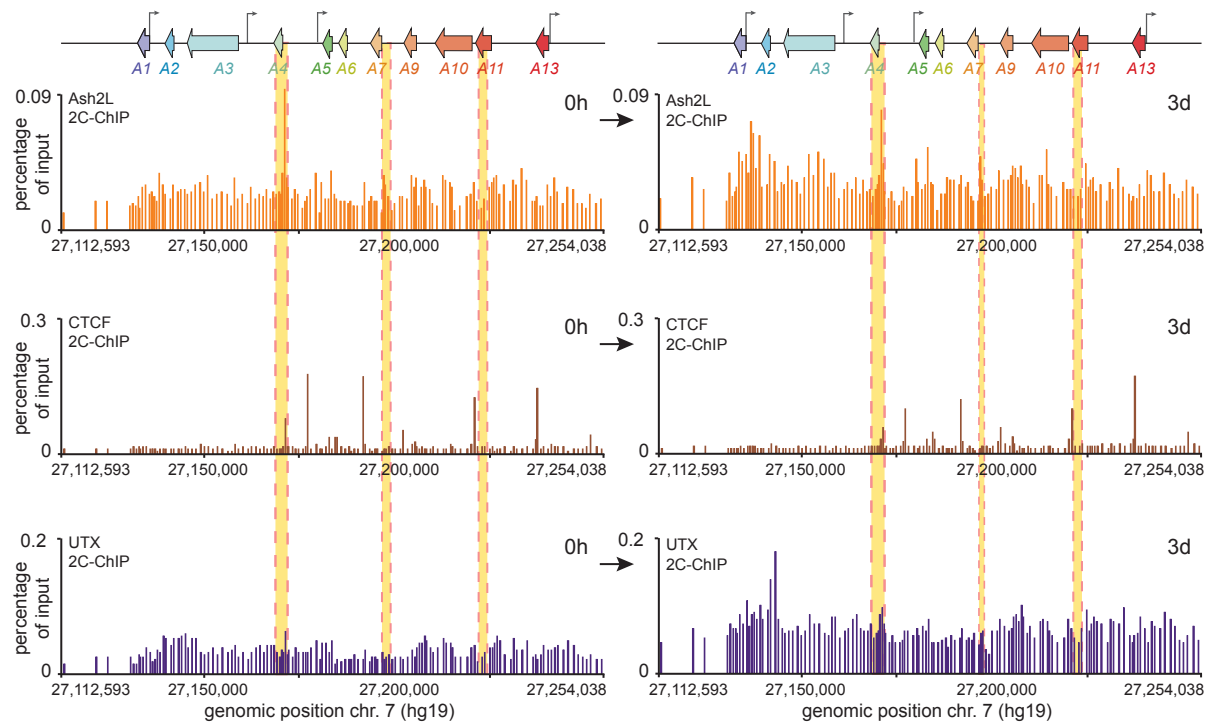

Supplement: Supplementary file 12 — Figure S6. 2C-ChIP results for Ash2L, CTCF, and UTX in set A. Data is displayed as outlined in Fig. 2f. The complete BED files including surrounding negative controls are in Additional file 6: BED file 31–36). (PDF 377 kb) [file 12864_2019_5532_MOESM12_ESM.pdf]

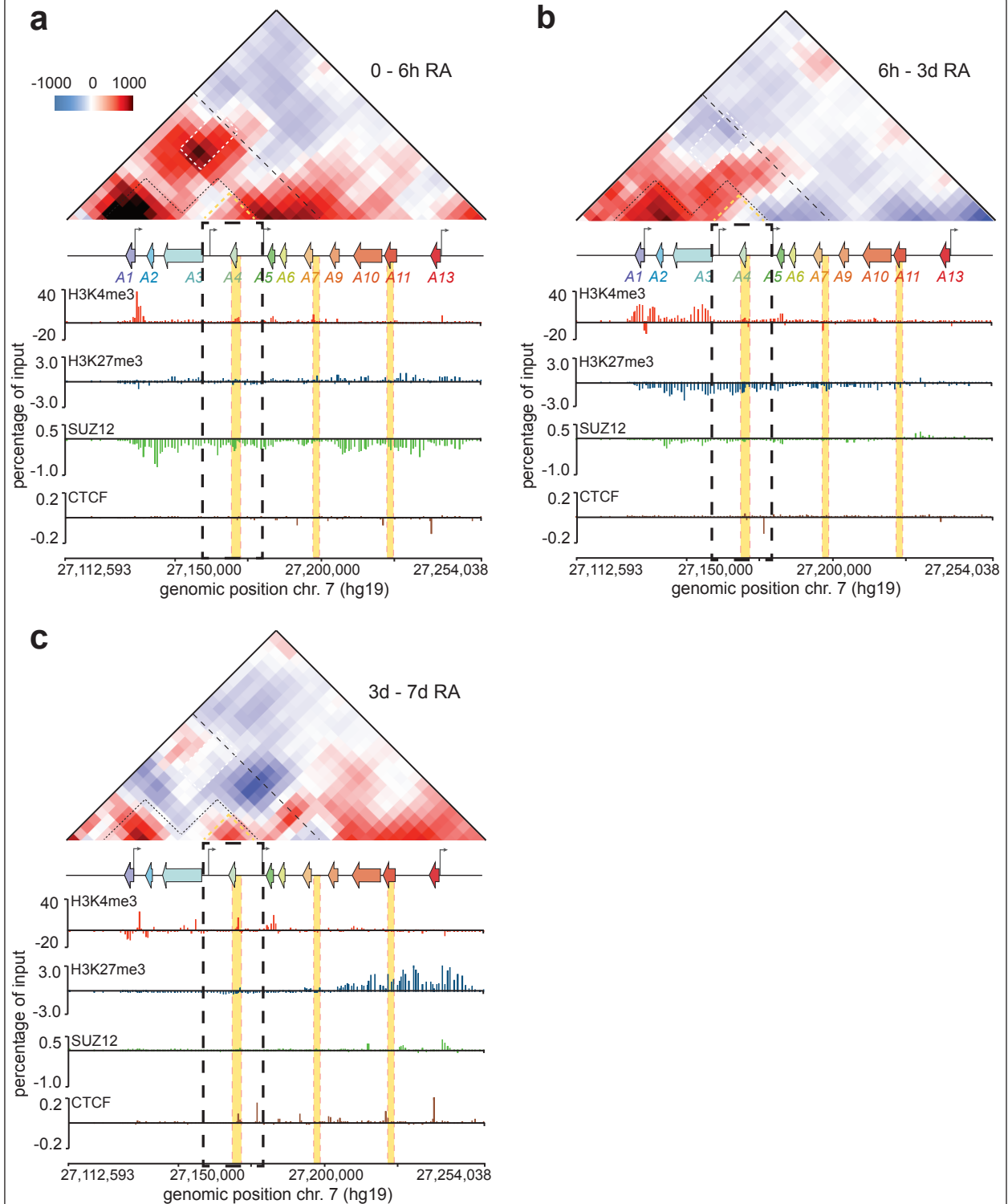

Supplement: Supplementary file 15 — Figure S7. RA-induced NT2-D1 differentiation is accompanied by extensive conformational and epigenomic changes along the HOXA gene cluster. Changes in the frequency of chromatin contacts occurring early after RA induction (a; 0 – 6 h RA), after 3 days (b; 6 h – 3d RA), or in the later phase of the time course (c; 3d – 7d) are shown in heatmap form. Heatmap values represent IF differences between later and earlier time points that are color-coded according to the scale in panel a, with blue indicating a loss of contact and red an interaction gain. Regions highlighted in heatmaps are as described in Fig. 5. Tracks under each heatmap represent corresponding changes in the levels of chromatin marks or bound proteins detected with 2C-ChIP. The dashed black box identifies a HOXA cluster region slow to lose H3K27me3 signal (PDF 572 kb) [file 12864_2019_5532_MOESM15_ESM.pdf]
